# Live fast, die young: Accelerated growth, mortality, and turnover in street trees

**DOI:** 10.1371/journal.pone.0215846

**Published:** 2019-05-08

**Authors:** Ian A. Smith, Victoria K. Dearborn, Lucy R. Hutyra

**Affiliations:** Boston University, Earth & Environment Department, Boston, Massachusetts, United States of America; Tennessee State University, UNITED STATES

## Abstract

Municipalities are embracing greening initiatives as a key strategy for improving urban sustainability and combatting the environmental impacts of expansive urbanization. Many greening initiatives include goals to increase urban canopy cover through tree planting, however, our understanding of street tree ecosystem dynamics is limited and our understanding of vegetation structure and function based on intact, rural forests does not apply well to urban ecosystems. In this study, we estimate size-specific growth, mortality, and planting rates in trees under municipal control, use a box model to forecast short-term changes in street tree aboveground carbon pools under several planting and management scenarios, and compare our findings to rural, forested systems. We find accelerated rates of carbon cycling in street trees with mean diameter growth rates nearly four times faster in Boston, MA, USA (0.78 ± 0.02 cm yr^-1^) than in rural forest stands of MA (0.21 ± 0.02 cm yr^-1^) and mean mortality rates more than double rural forested rates (3.06 ± 0.25% yr^-1^ in street trees; 1.41 ± 0.04% yr^-1^ in rural trees). Despite the enhanced growth of urban trees, high mortality losses result in a net loss of street tree carbon storage over time (-0.15 ± 0.09 Mg C ha^-1^ yr^-1^). Planting initiatives alone may not be sufficient to maintain or enhance canopy cover and biomass due to the unique demographics of urban ecosystems. Initiatives to aid in the establishment and preservation of tree health are central for increasing street tree canopy cover and maintaining/increasing carbon storage in vegetation. Strategic combinations of planting and maintenance will maximize the viability of greening initiatives as an effective climate mitigation tool.

## Introduction

By 2030, urban land cover could triple its 2000 extent [1], increasing anthropogenic pressure on regional ecosystems and the global climate. Urban vegetation provides ecosystem services that can help mitigate local urbanization impacts through reducing the urban heat island [2], reducing surface runoff [3], and improving mental health [4]. Given current trends in development and the suite of potential services urban forests provide, many city governments have undertaken major tree planting efforts [5]. However, cities have been dramatically understudied by ecologists [6] and there is an urgent need for scientific rigor to be applied to nature-based solutions in cities.

Despite the widely espoused benefits of urban trees [7], the role of urban vegetation in the carbon cycle remains uncertain [8]. Recent studies distinguish the unique ecosystem dynamics of the urban forest, describing considerable urban biogenic carbon fluxes [9,10] and large increases in the relative ecosystem productivity of vegetation with urbanization [11,12,13]. However, our knowledge of street tree carbon dynamics, including the balance of growth, mortality, and planting rates is data limited [14,15]. Given the high financial and environmental costs of planting and maintaining street trees [16,17,18] and the interest of policy makers in using urban tree planting for carbon credits [19,20], further research on species-level and size-specific demographics is needed to inform and assess the viability and carbon implications of greening initiatives.

Street trees, unlike vegetation on residential and commercial property, are planted and maintained under complex networks of multi-stakeholder governance [21], offering a unique opportunity for improving the urban environment. The management of street trees allows cities to be intentional with their planting and maintenance strategies to optimize their sustainability goals. Though rarely measured concurrently, there is a growing body of literature documenting street tree growth and mortality rates [22,23] and the parameterization of city-scale urban ecosystem models is starting to grow more sophisticated (e.g. i-Tree, 2017 [24], *cf*. [25]). Yet, in most larger scale ecosystem models and carbon accounting initiatives, the contributions of urban vegetation to the carbon cycle are entirely neglected or parameterized based on our knowledge of rural forests. For example, NASA’s MODIS Net Primary Productivity (e.g. MOD17) maps characterize nearly one-third of the state of Massachusetts as having zero biogenic carbon fluxes due to urbanization intensity [26], despite urban land covers in Massachusetts storing up to 42.7 Mg C ha^-1^ [10].

The role of street trees in the urban carbon cycle is complex as the environmental costs and benefits of street trees evolve over the course of a tree’s life, with emissions associated with establishment often precluding net greenhouse gas benefits in the early stages of a street tree’s life cycle. The carbon costs associated with nursery production, planting, irrigation, pruning, removal, and disposal are high [16]. Street trees must survive for several decades (26–33 years; [27]) to attain carbon neutrality. There is therefore a need for empirical estimations of current mortality, planting, and growth rates across species and size classes to develop efficient planting/maintenance strategies that maximize the proportion of street trees providing net carbon benefits.

In this study, we combine an existing 2005–06 census of street trees in Boston, Massachusetts, USA with a 2014 resurvey to assess recent trends in street tree carbon dynamics. We test the hypotheses that (1) decreasing mortality rates is more effective than increasing tree planting in improving the carbon balance of the urban forest and (2) carbon cycling is accelerated in street trees relative to rural forests, with high growth and mortality rates. We employ a box model to explore future scenarios for alternative planting and management strategies, offering quantitative insights into the carbon pools and fluxes associated with greening efforts over time. Additionally, we explore and contextualize the distinct ecology of the urban forest by quantitatively assessing the demographics and carbon dynamics of street trees relative to rural forests.

## Material and methods

### Study area

**Fig 1:**
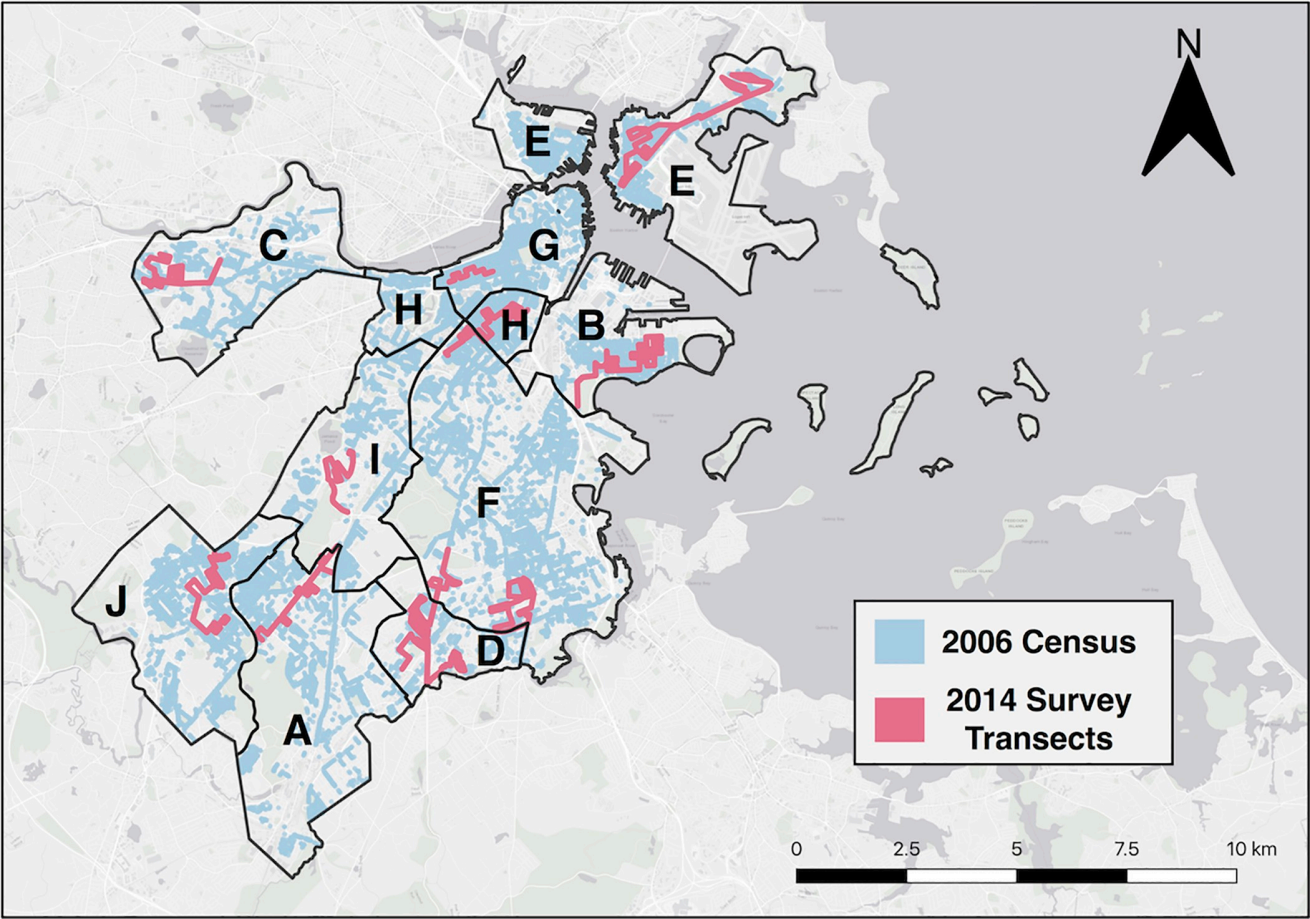
Census and survey transects. 2014 survey transects (red) overlaid on UEI surveyed trees (blue). Letters represent group ID’s corresponding to the 10 consolidated neighborhood groups. See S2 Table for neighborhood descriptions and statistics. Sources: Esri, Garmin, HERE, INCREMENT P, OpenStreetMap contributors, and the GIS community.

This study was conducted in the City of Boston, Massachusetts (Fig 1) and in a well-characterized, nearby rural forest located at the Harvard Forest Environmental Measurement Station (EMS) in Petersham, Massachusetts, USA. Massachusetts has a humid, continental climate characterized by cold, snowy winters and hot, humid summers. The mean annual precipitation rate is 105.6 cm yr^-1^ [28]. Soils in eastern Massachusetts are dominated by sand and silt loams [29]; however, almost all soils surrounding street trees in urban areas are highly disturbed and may not reflect the soil type of surrounding forested areas [30, 31]. Our rural reference sites are located at the Harvard Forest Long-Term Ecological Research site (~ 100 km west of Boston), an intact 85–120 year old [32] temperate forest dominated by red oak (*Quercus rubra*) and red maple (*Acer rubrum*).

### Street tree survey

During the summers of 2005 and 2006, the City of Boston commissioned a census of street trees [33], training 26 full-time interns and over 300 volunteers to collect basic tree demographics (S1 Table). A total of 38,765 trees were surveyed in the 2005–06 census conducted by Boston College’s Urban Ecology Institute, henceforth referred to as the UEI Survey. In the summer of 2014, we resampled approximately 10% of the original UEI Survey area to assess vegetation growth and demographic dynamics over time. For a policy relevant unit of analysis, the 2014 survey was stratified across the Boston Planning and Development Agency neighborhoods, excluding the Harbor Islands, which were aggregated into 10 neighborhood groups based on proximity (Fig 1). To capture local variability within and across neighborhoods the sampling included (1) main thoroughfares with consistent daily traffic; (2) less trafficked side streets; and (3) a range of soil cover types and pruning intensities. For the purposes of statistical analysis, we define a “plot” as one side of a street block. We surveyed a total of 3,500 individual trees across 590 plots, along 10 transects corresponding to the 10 neighborhood groups (Fig 1).

In 2014, a two-person team walked the length of each transect, surveying each street tree. Following the methods of the UEI survey, diameter at breast height (DBH, 1.37 m) was measured to the nearest 0.1 cm using DBH tapes. Latitude and longitude waypoint coordinates were recorded with a handheld GPS unit. Trees were identified to species, except for some exotic ornamental trees which were identified to genus. Any tree unequivocally identified in the field as a UEI Survey tree was assigned its respective UEI Survey ID *in situ*; however, most trees were matched post-survey based on coordinate location, species, and size. Citizen science urban tree inventories can introduce more error into measurements than those conducted by experts [34]; to ensure individual trees were accurately compared between the two surveys and to minimize error associated with differences in DBH tape position between the two surveys, a rigorous data quality control method was employed (S3 Table). Boston generally plants street trees that are approximately 5 cm DBH (M Ford-Diamond, personal communication, 2014); reported statistics are for trees larger than 5 cm DBH unless otherwise noted. Trees present in the UEI Survey that were no longer present in the 2014 resurvey were considered lost to mortality or removal (henceforth referred to as “mortality”). Trees that existed in the resurvey which had not previously existed during the UEI Survey were considered planted recruits.

### Harvard forest data processing

In 1993, 40 circular 10 m radius plots were installed within the footprint of the Harvard Forest EMS eddy flux tower (42.537755°N, -72.171478°W; [35]). The EMS plots contain 25 years of detailed ecological data including annual measurements of DBH, mortality, and recruitment. We use these datasets to compare our urban observations to a nearby rural forest. Dominant common species across our urban and rural sites include *Quercus rubra* and *Acer rubrum*.

In our analysis, we only use data from 34 of the original 40 plots at Harvard Forest as 3 plots were flooded by a beaver dam and 3 plots were selectively logged in 2001. Additionally, we only report biometric data from 2006–2014 to coincide with the timeframe of street tree sampling, but the patterns observed are present across the full time series (i.e., 1993–2017). The minimum DBH requirement for inclusion in the dataset was 10 cm and trees exceeding 10 cm DBH that were <10 cm DBH in the previous year were considered recruits.

The dynamics of open-grown street trees will clearly differ from those of a mature, even-aged, second growth forest with a closed canopy. Nonetheless, this comparison is valuable to highlight contrasts in the ecosystem dynamics. Most landscape-scale modeling efforts to estimate biogenic carbon fluxes do not account for the unique growing conditions of the urban environment and treat urban vegetation as the native background vegetation. Our comparison contextualizes the relative magnitude of urban street tree fluxes and examines the validity of landscape-scale model assumptions.

### Statistical analysis and model development

For Boston analyses, tree mortality was calculated as the number of trees that were alive in 2006 and dead or removed by 2014 divided by the number of 2006 trees in a given plot [36]. This is a conservative estimate as we did not account for “ghost mortalities” [15]—trees that may have been planted, died, and been removed between the two surveys in 2006 and 2014. The recruitment rate, also called in-growth, was calculated as the number of trees planted since 2006 divided by the number of trees present in 2006 in a given plot [23]. Growth rates were calculated as the difference between 2014 DBH and 2006 DBH. Annualized estimates were calculated by dividing by the 8-year survey interval (2006–2014).

For Harvard Forest analyses, tree mortality was calculated for each year in the study period as the number of dead trees in a plot in a given year that were alive in the previous year, divided by the total number of living trees in the plot in the previous year. The recruitment rate was calculated as the number of recruits in a plot in a given year divided by the total number of living trees in the plot in the previous year. Reported mortality and recruitment rates represent the average annual rates from 2006–2014. Growth rates were calculated as the difference between 2014 DBH and 2006 DBH and annualized by dividing by the 8-year study interval.

Rural forest allometric equations may be less representative when used to estimate open-grown or pruned urban tree biomass [37], therefore species-specific allometries for open grown urban trees [38] were applied to estimate surveyed tree biomass in Boston. In cases where a species-specific allometry was not available, we applied the McPherson et al. [38] urban general broadleaf equation. The street tree biomass estimates presented in this study are included for comparative purposes and are not meant to serve as precise enumerations of biomass stocks. Previous research in Boston found the mean carbon storage in all trees to be 28.8 Mg C ha^-1^ [39]. For consistency with other publications, the Harvard Forest biomass estimates are based on the site recommended species-specific allometric equations [40].

Tree biomass per plot area is presented as Mg C ha^-1^, assuming 50% of the biomass is carbon. Stem density per plot area is presented as stems ha^-1^. The Boston plots had variable area, defined as the public land area (middle of the street to the edge of the sidewalk), and was delineated manually for each of the 590 plots in Google Earth Pro (Version 7.3) by drawing polygons encompassing half of the road and all of the sidewalk.

A box model representing the aboveground carbon stocks and fluxes was developed following the methods and structure of Pyle et al. [41]. The model was used to assess short-term tendencies of both street trees and rural, forested trees. Each size class accumulates biomass within the class, transfers biomass into the next larger size class, and dies, at rates approximated using 1st-order kinetics (S1 Fig). Trees <10 cm DBH were omitted from the street tree box model for consistency with Harvard Forest data. All model coefficients were empirically determined from direct measurements. Alternative planting and management scenarios in Boston were simulated by increasing or decreasing the planting and mortality parameters relative to their current means. All models were run 1000 times with initial model parameters for growth, recruitment/planting, and mortality stochastically selected from a Gaussian distribution around their observed means.

All statistical analysis and modeling was completed using R Statistical Software 3.4 (R Core Team 2016). All reported mean values are weighted by plot area and errors are 95% confidence intervals with the sample plot as the unit of replication. Error estimates include field sampling, but do not include allometric or spatial scaling errors. Box model estimates reflect 95% confidence intervals based on 1000 model realizations.

## Results

### Growth, mortality, & planting

**Fig 2:**
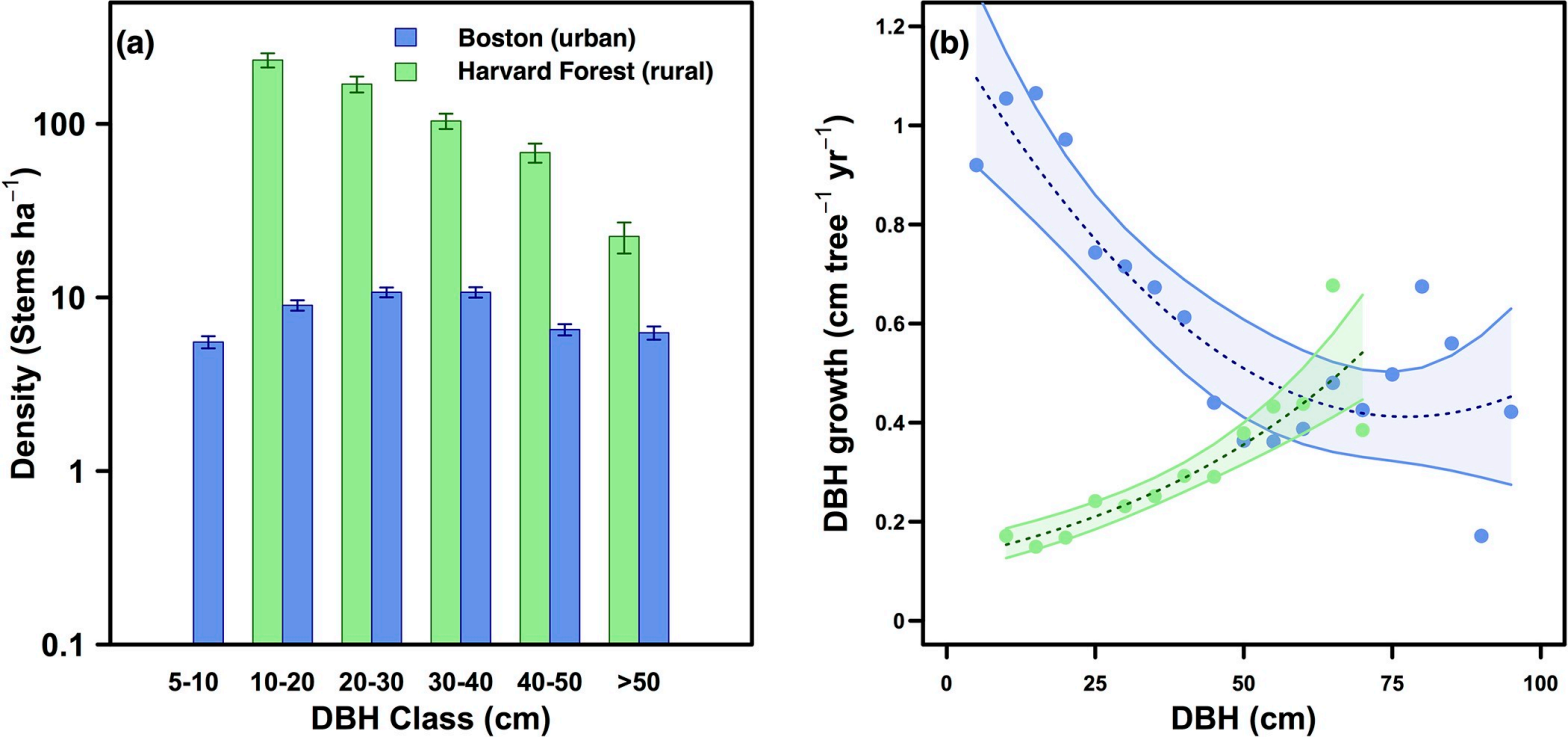
Stem density & growth. a) Stem density in 2014 (stems ha^-1^) shown on a log scale. b) DBH growth (cm tree^-1^ yr^-1^) in Boston and at Harvard Forest, binned by 5 cm DBH classes. A 2^nd^-order polynomial regression model and exponential growth model with 95% confidence intervals are fit to the observations in Boston and Harvard Forest, respectively (Boston: R^2^ = 0.75, p-value < .001; HF: R^2^ = 0.87, p-value < .001).

Size-dependent vegetation structure and demographic characteristics varied considerably between urban street trees in Boston and the rural Harvard Forest (Fig 2). The Boston stem density distribution was largely uniform while Harvard Forest showed a size-density decline (Fig 2A). Street tree growth rates (0.8 ± 0.02 cm tree^-1^ yr^-1^) were higher on average than rural forested trees (0.2 ± 0.02 cm tree^-1^ yr^-1^; Fig 2B) with resultant mean street tree basal area increment values (36.8 ± 2.2 cm^2^ tree^-1^ yr^-1^) more than three times higher than rural forested values (10.2 ± 1.0 cm^2^ tree^-1^ yr^-1^). The mean diameter of street trees was slightly larger (DBH = 29.6 ± 1.1 cm) than rural forested trees (DBH 26.4 ± 1.2 cm). Street trees <25 cm DBH in Boston grew approximately 5 times faster than trees of the same size class at Harvard Forest, but the two sites showed opposite dynamics in growth rates as a function of size with urban growth rates declining exponentially with size (Fig 2B). Faster urban growth rates were also observed in dominant individual species across the region with *Acer rubrum* and *Quercus rubra* >10 cm DBH growing 362% and 148% faster, respectively, in the urban environment (Table 1).

**Table 1:** Growth and mortality rates in Boston street trees (Urban) and Harvard Forest trees (Rural). Quercus rubra (urban n = 123; rural n = 126) and Acer rubrum (urban n = 167; rural n = 267) represent shared dominant native species between sites. For consistent comparison with Harvard Forest data, reported numbers for Acer rubrum and Quercus rubra only include trees > 10 cm DBH.

		Mean 2006 DBH (cm)	DBH growth (cm stem^-1^ yr^-1^)	Mortality rate (% stems yr^-1^)
**All Trees**	Rural	26.4 ± 1.2	0.21 ± 0.02	1.41 ± 0.04
	Urban	29.6 ± 1.1	0.78 ± 0.02	3.06 ± 0.17
***Quercus rubra***	Rural	36.2 ± 2.1	0.33 ± 0.04	0.12 ± 0.07
	Urban	37.9 ± 6.5	0.82 ± 0.19	4.82 ± 1.11
***Acer rubrum***	Rural	19.2 ± 0.9	0.21 ± 0.03	1.08 ± 0.21
	Urban	19.3 ± 1.7	0.97 ± 0.13	2.86 ± 0.62

**Fig 3:**
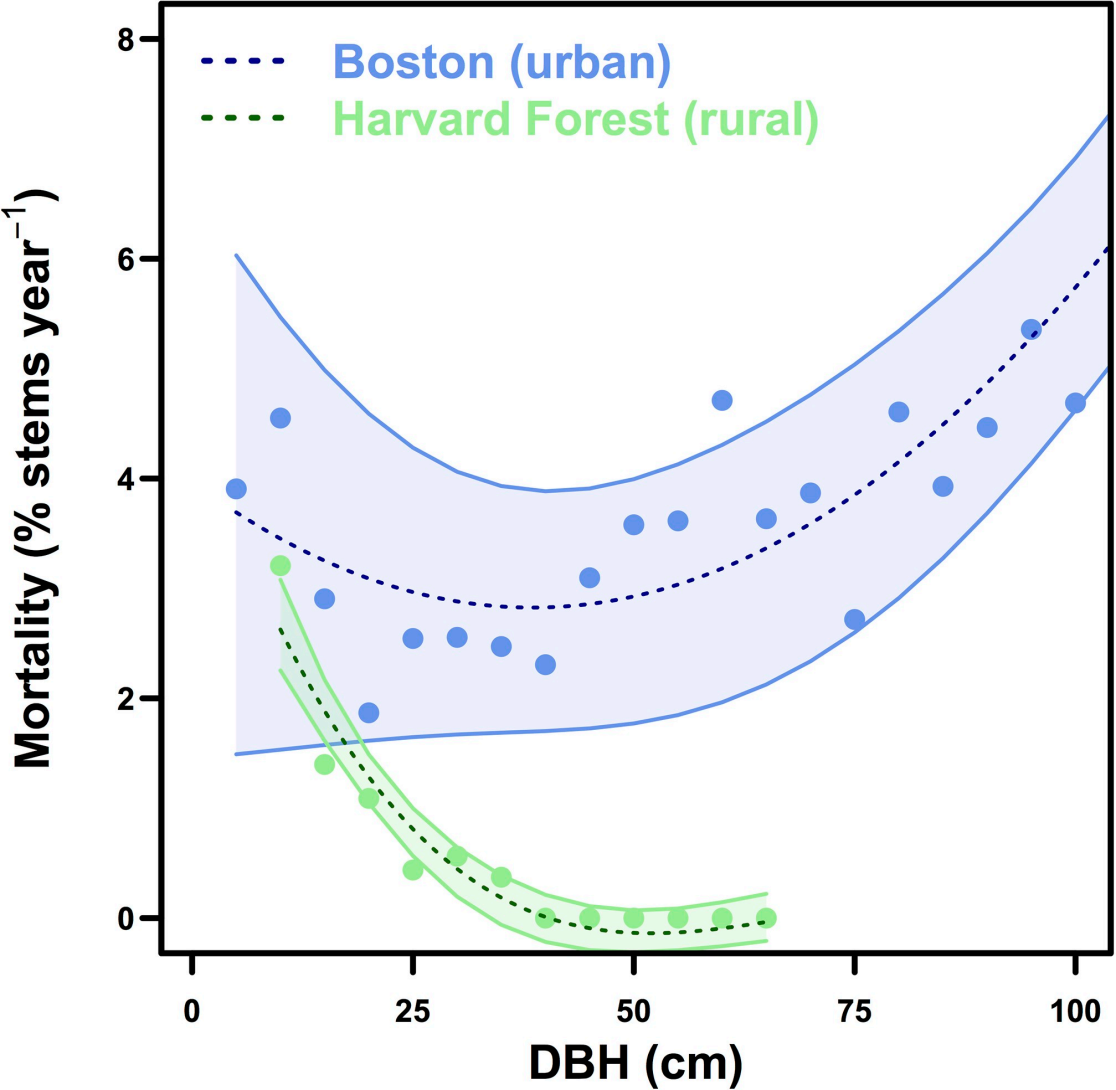
Mortality. Mortality rates in Boston and at Harvard Forest (% stems yr^-1^), binned by DBH. 2^nd^-order polynomial regression models with 95% confidence interval are fit to the observations (Boston: R^2^ = 0.63, p-value < .001; HF: R^2^ = 0.92, p-value < .001).

Mean street tree mortality rates (3.1 ± 0.2% stems yr^-1^) between 2006 and 2014 were more than double non-urban rates (1.4 ± 0.04% yr^-1^), with stark differences across size classes. While mortality rates of trees <20 cm were high in both locations, the mortality rate of rural trees declined exponentially with tree size. In contrast, we observe a u-shaped mortality curve in urban street trees and the highest rates of mortality in the smallest and largest size classes (Fig 3).

The rates of planting (2.9 ± 0.6%) nearly equaled the rates of mortality in urban street trees, but the biomass changes associated with these two processes do not cancel due to differences in size between the trees being planted and those dying. City planners in Boston generally plant street trees with a DBH of ~5 cm (M Ford-Diamond, personal communication, 2014); in contrast, the mean DBH of the street trees that died during the study was 30.5 ± 2.1 cm. The biomass gains associated with planting were only 0.02 ± 0.003 Mg C ha^-1^ yr^-1^ and the losses from mortality were 0.84 ± 0.2 Mg C ha^-1^ yr^-1^. The total live stem frequency of street trees across the study area only decreased by 141 stems (4%) over the 8-year study interval (n_2006_ = 3448 and n_2014_ = 3307), with 75% of the trees in 2006 surviving to 2014 and 674 new stems planted.

Mortality varied substantially by genera in street trees and was higher than non-urban trees of the same genera (Table 1). Genera-specific street tree mortality rates ranged from 1.6 ± 0.6% yr^-1^ (*Gleditsia*; n_2014_ = 468) to 5.6 ± 1.4% stems yr^-1^ (*Pyrus*; n_2014_ = 216; S4 Table). During the study interval, policy changes occurred regarding preferred species for planting. The City of Boston’s most recent list of Parks Department Approved Street Trees [42] no longer includes several of the most common genera found in our survey (*Fraxinus*, *Pyrus*, and *Platanus*).

**Fig 4:**
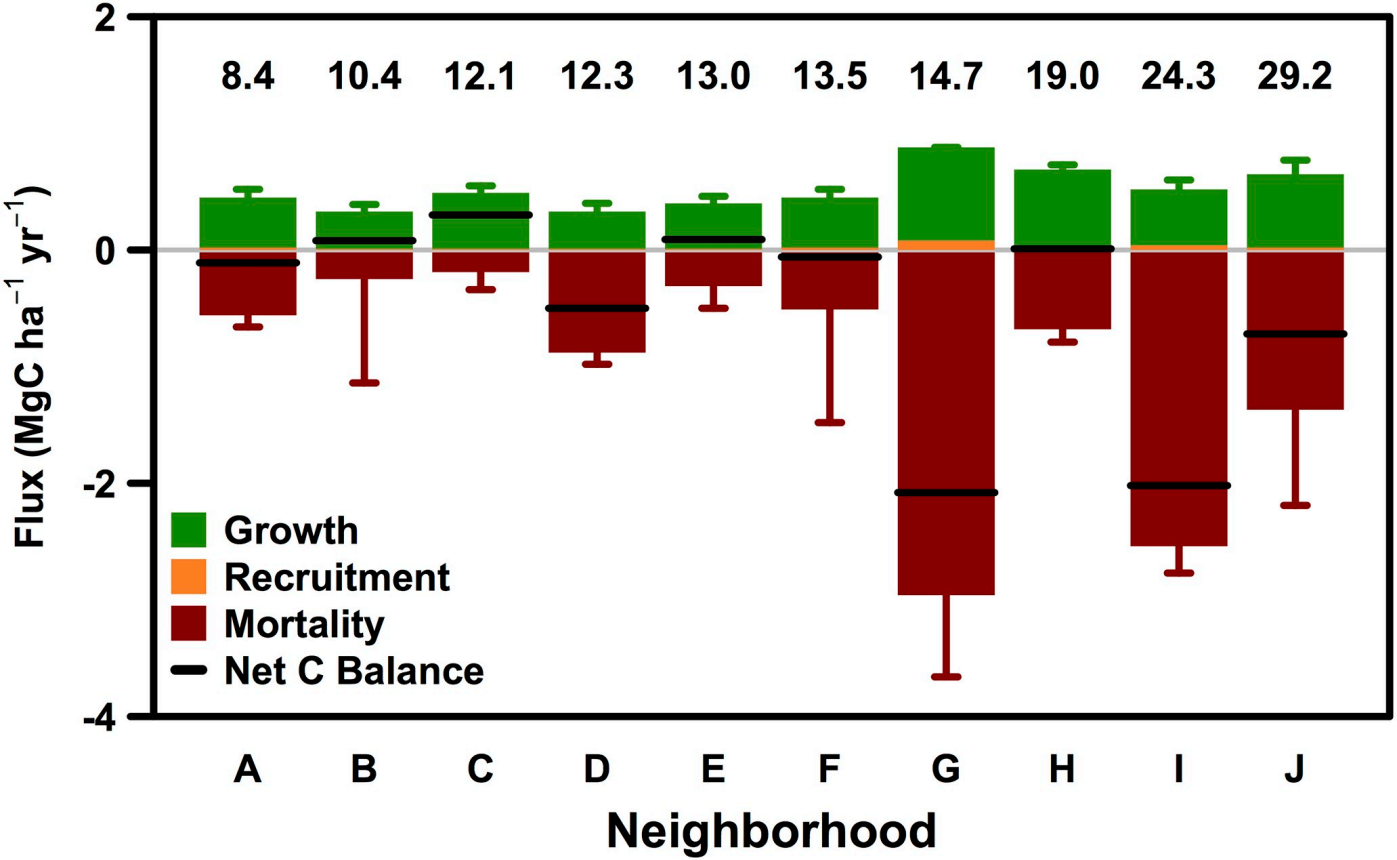
Neighborhood C balance. a) Neighborhood group net carbon flux (Mg C ha^-1^ yr^-1^) throughout the City of Boston as a balance of biomass change due to mortality, recruitment, and tree growth between 2006 and 2014. Error bars represent standard error. Numbers above bars represent 2014 biomass stock (Mg C ha^-1^). See S2 Table for neighborhood descriptions and statistics.

The mean carbon density in Boston street trees was 15.6 ± 1.9 Mg C ha^-1^ in 2006 and 14.3 ± 1.5 Mg C ha^-1^ in 2014. The net change in aboveground carbon balance for street trees was estimated as the sum of growth and planting, less losses from mortality with negative values corresponding to net losses of carbon. The annual net carbon balance across 590 plots was estimated to be slightly negative at -0.15 ± 0.09 Mg C ha^-1^ yr^-1^. The carbon balance across neighborhoods ranged from -2.08 ± 0.84 Mg C ha^-1^ yr^-1^ to 0.30 ± 0.20 Mg C ha^-1^ yr^-1^ (Fig 4).

Neighborhood Groups G, I, and J experienced large carbon losses associated with the mortality of very large, old trees as these neighborhoods had 3 of the 4 largest standing biomass stocks (Fig 4) and average diameters in 2014. Across neighborhoods, street tree biomass ranged from 8.4 ± 2.3 Mg C ha^-1^ in Group A to 29.2 ± 11.1 Mg C ha^-1^ in Group J (Fig 4).

### Box model and management scenarios

**Fig 5:**
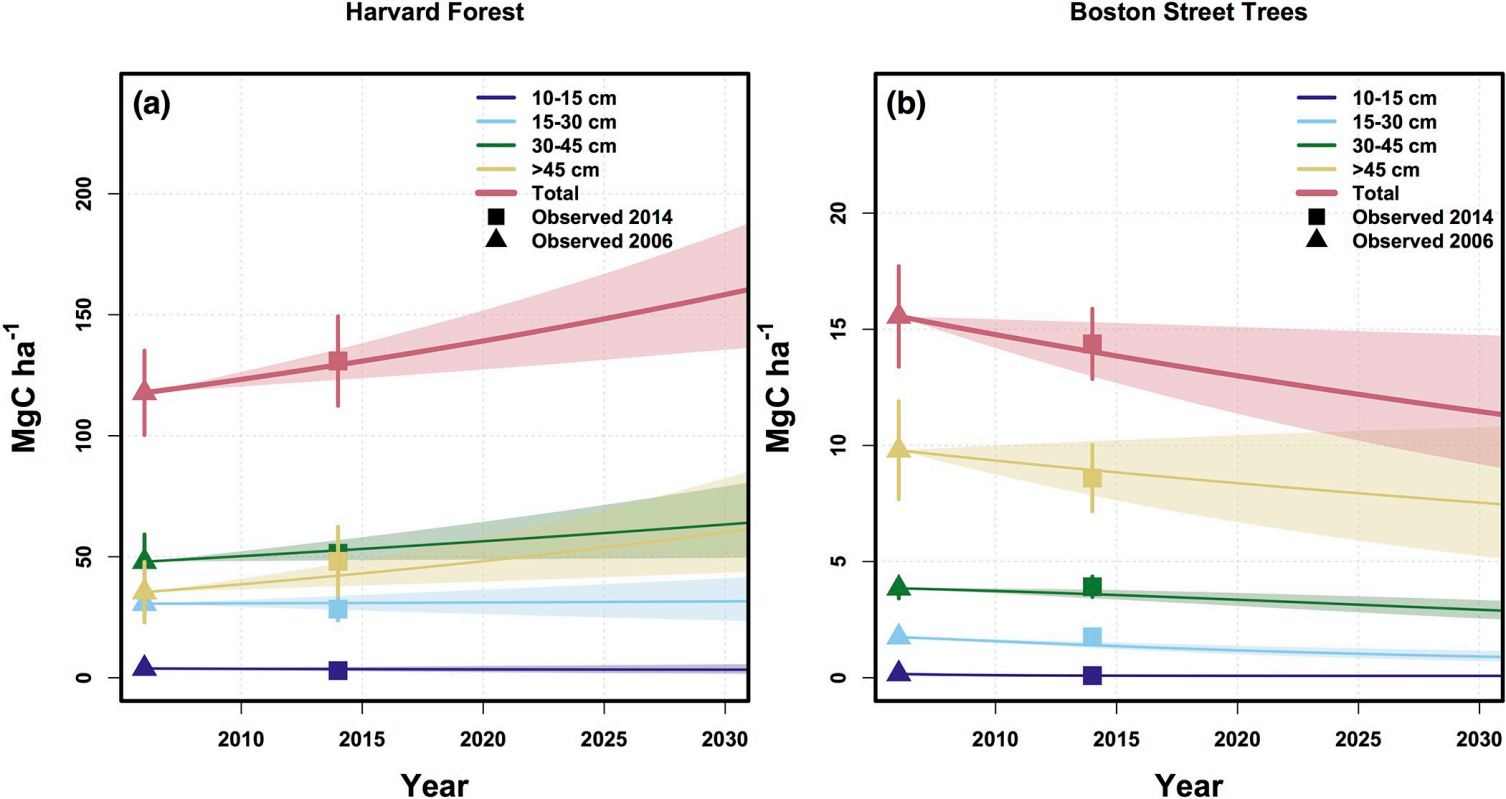
Projected C storage. Projected aboveground carbon pools at rural Harvard Forest (a) and in urban Boston street trees (b) between 2006 and 2030. Triangles represent 2006 observations ± 95% CI and squares represent 2014 observations ± 95% CI. Pools are broken up by size class with the total carbon storage represented in red. Models were run 1000 times and shaded polygons represent the middle 95% of model observations. Model structure and parameters can be found in S1 Fig.

Based on observed planting, mortality, and growth rates and a business as usual scenario, our box model estimates that by 2030 the mean street tree biomass density in Boston will decline by 26% from 15.6 ± 1.9 Mg C ha^-1^ in 2006 to 11.5 (95% CI: 9.0–14.5) Mg C ha^-1^ in 2030 (Fig 5B). In contrast, the biomass density at Harvard Forest is projected to increase 34% above the 2006 biomass density of 117.7 ± 9.0 Mg C ha^-1^ to 157.9 (95% CI: 135.0–185.4) Mg C ha^-1^ by 2030 (Fig 5A). The projected increase in carbon storage at Harvard Forest is largely driven by the low mortality rate of large trees, whereas the projected decline in street tree carbon storage is the result of large biomass losses from mortality outweighing high growth rates of newly planted and smaller living trees.

**Fig 6:**
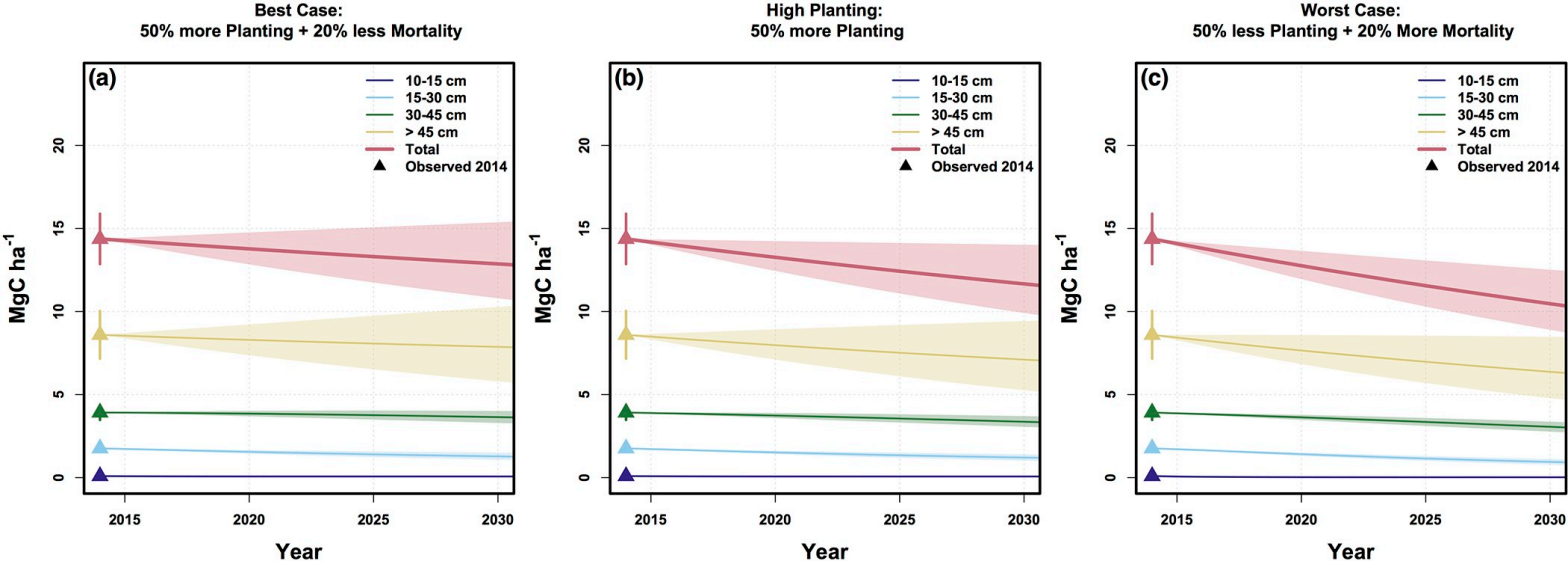
Scenarios. Projected aboveground carbon pools in Boston street trees between 2014 and 2030 under various changes to current planting and maintenance regimes. Triangles represent 2014 observations with a 95% confidence interval. Pools are broken up by size class with the total carbon storage represented in red. Models were run 1000 times and shaded polygons represent the middle 95% of model observations.

Under our best-case scenario in which planting rates increase 50% and tree maintenance increases such that mortality rates decrease 20% across all size classes, the street tree biomass density is projected to be relatively stable at 12.9 (95% CI: 10.8–15.1) Mg C ha^-1^ through 2030 (Fig 6A). In contrast, under a scenario in which planting increases by 50% and nothing is done to address mortality rates, the C density is still projected to decrease to 11.7 (95% CI: 10.2–13.9) Mg C ha^-1^ by 2030 (Fig 6B), which is nearly identical to the projected 2030 biomass density under the business as usual scenario (Fig 5B). If planting rates decrease by 50% and nothing is done to address mortality rates, the street tree carbon density is projected to decrease by 32% below the 2006 carbon density to 10.5 (95% CI: 8.7–12.4) Mg C ha^-1^ by 2030 (Fig 6C).

## Discussion

Urban greening efforts often focus on planting to achieve increases in canopy cover, but despite high planting and growth rates in many urban areas of the US, there has been a decrease in urban canopy cover over time [43]. In New York City, 26.2% of street trees died within 9 years of planting [44] and an analysis of 11 previous studies from across the globe suggests that the typical street tree population half-life (the time at which cumulative survivorship is 50%) is only 13–20 years [45]. In Milwaukee, WI and Denver, CO, more than 28,000 trees were lost in 5 years with more stems lost in older neighborhoods with higher canopy cover [46]. In some cases, high planting rates have led to net increases in street tree population [15], however, this pattern does not necessarily mean that cities are succeeding in increasing biomass or canopy cover. Removal of large trees for (re)development [47] is often justified by replanting several seedlings in its place [48], but high mortality rates and the time required for a seedling to reach full stature can result in a sustained canopy decline for many years.

Our study takes a novel approach to assessing urban tree dynamics by examining the balance of tree planting, mortality, and growth and quantifying the net changes in biomass, allowing for a quantification of biomass loss via mortality and biomass gain through planting and growth. Further, we show that our understanding of vegetation structure and function based on intact, rural forests does not apply well to urban ecosystems. Urban vs. rural comparisons are always challenging due to a myriad of confounding factors, however, such comparisons have been a primary framing for urban ecology research for decades [49]. The form of urban areas makes it nearly impossible to have fully comparable study sites and survey methods. Here, we carefully compare the dynamics of nearby urban and rural trees, highlighting the observed differences in trees of the same species and size during a consistent time period. We find higher turnover in urban street trees, with higher rates of growth and mortality relative to rural forests, which is consistent with modeling studies suggesting an accelerated rate of carbon cycling in urban vegetation [10].

**Fig 7:**
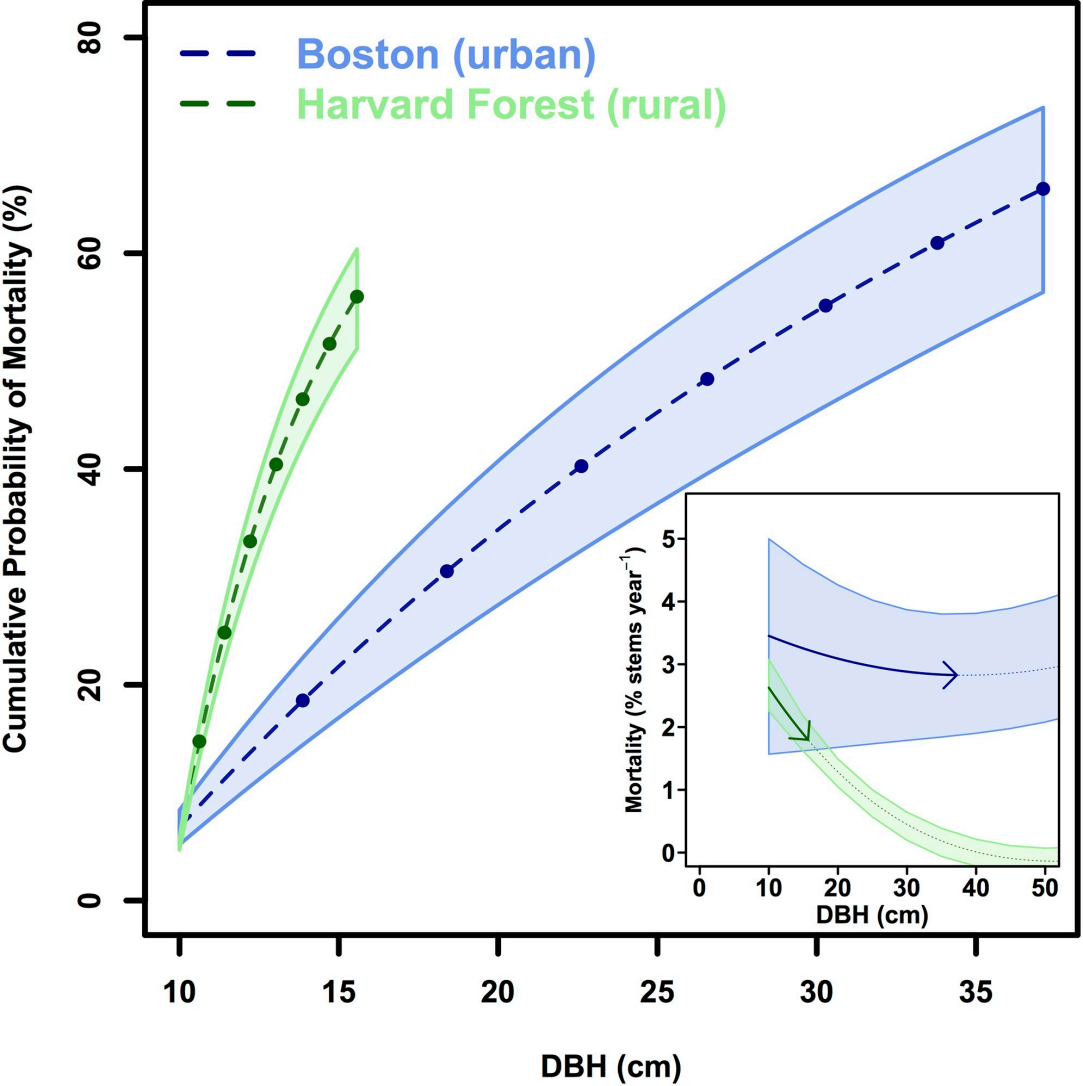
Mortality probability. Cumulative probability of mortality (%) vs. DBH (cm) for a 10 cm DBH tree in an urban vs. rural environment over a 35 year period. Points represent 5 year increments. Inset: Annual mortality rates (%) vs. DBH (cm) in Boston and at the Harvard Forest. Solid arrows represent the trajectory of the 10 cm DBH tree after 35 years.

If we consider a 10 cm DBH tree growing in an urban streetscape and in an intact rural forest, the urban tree would grow to be 37.7 ± 2.9 cm in diameter (Fig 7) and store 279.2–398.7 kg C after 35 years. The same tree in an intact rural forest would grow to be less than half of the size of the urban tree (15.7 ± 0.8 cm DBH; Fig 7) after 35 years and would store an order of magnitude less carbon (34.3–44.2 kg C). However, the probability of the urban tree surviving 35 years is only 35.1 ± 8.6%, while the rural tree has a 44.2 ± 4.5% chance of survival (Fig 7). Urban and rural tree mortality rates are similar for trees less than 20 cm in DBH (Fig 7: inset), but the urban growth rates are enhanced for trees less than 50 cm in DBH (Fig 2B).

Differences in both growing conditions and management between urban and rural areas determine the ecosystem carbon dynamics. In young street trees, opportunities for growth include more light availability with open growth conditions, elevated carbon dioxide concentrations (street level CO_2_ mixing ratios typically >500 ppm; [50]), enhanced reactive atmospheric nitrogen deposition (atmospheric N inputs in urban sites more than double rural sites; [51,52]), and an extended growing season relative to a rural forest (18–22 days longer in urban Boston relative to adjacent rural areas; [53]). While the urban heat island may push trees beyond their photosynthetically optimal temperatures [10,54], established urban trees can tap both water and sewer lines for additional resources [55]. Initially, young trees in both urban and rural environments experience high rates of mortality, but for different reasons. As urban street trees are planted, their early risks for mortality are associated with initial root establishment and access to water during that critical period. In contrast, for naturally regenerated small trees, survival and productivity are a function of competition for light and nutrients. Once a rural, forested tree reaches establishment with access to the canopy, the annual risk of mortality decreases substantially (Fig 3), while urban street trees encounter new size-specific risks such as root space limitation, excessive pruning, and anthropogenic removal due to hazard risk [56,57]. Street tree maintenance and irrigation can lower the risk of mortality [17,18,58,59], but the very maintenance practices that facilitate survival and rapid street tree carbon sequestration have high carbon costs themselves [27]. In contrast, slower growing rural trees typically have much lower maintenance carbon costs on a per tree basis.

This analysis highlights structural and functional differences between urban and rural ecosystem dynamics, with implications for ecosystem modeling at various scales. Urban areas are projected to continue expanding in the future [1], altering the structure and function of the environments in which they replace [60]. Improving our understanding of urban ecosystem dynamics and incorporating distinct urban biogenic fluxes into landscape scale ecosystem models and carbon budgets are critical for accurately characterizing the urban carbon cycle and urban ecosystem services.

In city-scale ecosystem models that are designed to capture the accelerated carbon cycling of urban trees (e.g. i-Tree Eco), demographic approaches that consider size- and species-specific growth and mortality rates will improve the accuracy of carbon uptake and storage estimates. While the average growth (0.8 ± 0.02 cm tree^-1^ yr^-1^) and mortality (3.1 ± 0.2% stems yr^-1^) rates found in this study agree well to the default parameters in i-Tree Eco v6.0 (0.8 cm tree^-1^ yr^-1^; 3.0% stems yr^-1^; [24]), we observe very large variation in these variables as a function of tree size.

### Coupled biogeochemistry & management

The aboveground carbon balance of street trees is only one component of the urban carbon cycle. The carbon balance of an ecosystem as a whole consists of the balance between vegetative growth, mortality, and respiration dynamics. In this analysis, we do not consider the role of respiration associated with urban street trees or carbon uptake by trees on private/commercial properties and urban woodlands. Urban land management practices, such as landscaping, fertilizing, and mulching, lead to soil respiration rates that can be more than double rural forest rates [61] and the growth enhancement that we observed here is not unique to street trees, but has also been observed in urban forest fragments [11, 13]. Model estimates that account for these dynamics highlight the importance of urban vegetation across landscapes and show that despite high rates of soil respiration, the reduced extent of pervious surface area in cities, coupled with stimulated net primary productivity, may allow urban vegetation to operate as a net carbon sink [10].

The ecosystem dynamics of urban trees likely vary by biome and our observations in Boston may not be representative of all cities. For example, urban canopy cover was estimated to range from 9.6% in Denver, CO to 53.9% in Atlanta, GA compared to 28.5% in Boston, MA [43]. Urban canopy cover decline was estimated to be as fast as 1120 ha yr^-1^ in New Orleans, LA, compared to 20 ha yr^-1^ in Boston, MA [43]. Local climate differentiates urban forest structure [62] and in this study, we describe the dynamics of trees in a mesic city that is generally not water limited and not excessively hot. In hotter, more arid cities, where water is likely a limiting agent, growth and mortality trends, and their associated ecosystem services may differ. Trees in arid cities require more frequent irrigation and the management of street trees could potentially stress local water supplies. Tropical environments, where urban tree growth can be rapid, are under-studied in the literature [63], but may also behave differently than assumed. Additional research across biomes is needed to assess the viability of greening initiatives in cities that differ substantially from temperate, mesic ecosystems.

### Management scenarios and implications for greening initiatives

In Boston, contractors offer street tree warranties and now provide basic maintenance (watering trees once every two weeks) and guarantees for 2 years (replacing any trees that die within 2 years of planting) [64]. Beyond the warranty period, street trees face the risks of growing in the urban environment, reducing the probability of successful tree establishment. In the case of Boston, tree planting efforts alone seem insufficient to increase canopy cover. In fact, we observe the highest rates of mortality and carbon loss in the neighborhoods with the most biomass (Fig 4), suggesting that policy makers should consider alternative strategies to reduce mortality rates of large trees. Some municipalities have ordinances to protect large trees from removal [48] and urban tree canopy cover has been observed to increase following the implementation of stringent tree protection regulations [65]. Our analysis of the impact of various planting and maintenance strategies on street tree biomass (Fig 6) shows that due to the difficulty of young tree establishment and high mortality risk of large street trees, municipal action to lower mortality rates can have a much larger impact on the total biomass of street trees than increasing the planting rate alone.

In this study, we find that through rapid growth rates, street trees have the ability to sequester carbon and potentially provide other ecosystem services, such as evaporative cooling [66], more efficiently than rural trees. Currently, these benefits are not fully realized due to the high mortality suffered by street trees. Diversified greening efforts that not only plant more trees, but also aid in the establishment of small trees and promote the health and maintenance of larger trees, could simultaneously abate urban mortality and maximize street tree ecosystem services. With cities at the forefront of implementing actionable climate mitigation policies to offset rising temperatures and atmospheric carbon dioxide concentrations, there is an urgent need to revise current strategies behind greening campaigns to capitalize on the unrealized, abundant ecosystem services provided by the urban canopy.

## Supporting information

S1 Table 2006 UEI survey data collection summary. For each tree, information was recorded in each category with the level of specificity described. In our study, units were converted to SI for statistical analysis.
(PDF)

S2 Table Neighborhood group statistics. For each of the aggregated study neighborhoods groups, per capita income, standing 2014 biomass, and net carbon balance are noted. Groups are assigned identifying letters in order of standing biomass.
(PDF)

S3 Table Data quality. UEI and 2014 survey data quality methodology.
(PDF)

S4 Table Mortality rates. Genus-specific mortality rates ± SE of the 10 most common genera in Boston between 2006 and 2014.
(PDF)

S1 Fig Box model. Structure and parameters for box models forecasting short term aboveground carbon pools in Boston street trees (top) and Harvard Forest trees (bottom). Boxes represent pools (Mg C ha^-1^) and arrows represent fluxes (Mg C ha^-1^ yr^-1^). Errors are 95% confidence intervals.
(PDF)
