# Supplementary material for: Live fast, die young: Accelerated growth, mortality, and turnover in street trees
Source: PLoS One. 2019 May 8;14(5):e0215846. doi: 10.1371/journal.pone.0215846 (PMC6505744; doi:10.1371/journal.pone.0215846)
Supplement: S1 Table — For each tree, information was recorded in each category with the level of specificity described. In our study, units were converted to SI for statistical analysis. (PDF) [file pone.0215846.s001.pdf]

Data	Description
Species name	Principle unit for identification. Fruit trees commonly given genus only.
Common name	Identification, if known
Height	Categorical estimation of total height of tree: less than 12 ft, 16-25 ft, 26-35 ft, 35-50 ft
Trunk segments	Numeric to identify the number of trunk segments
Diameter at breast height	Numeric diameter of the trunk (in) measured at a height of 4.5 ft
Crown spread	Categorical estimation of crown diameter: less than 12 ft 16-25 ft, 26-35 ft, 35-50 ft
Height to crown	Numerical measurement of the height from the ground to the start of the crown (ft)
Health	Categorical rating of health:<br>Poor = >30% dead branches, sparse foliage, severe wounds/cavities on trunk, conks/mushrooms<br>Fair = 10-30% dead branches, thinning/reduced foliage, non-severe wounds/cavities, no conks/mushrooms<br>Good = <10% dead branches, normal foliage, no open wounds/cavities, no conks/mushrooms
Dead rating	Categorical rating of dead branches: <10%, 10-30%, >30%
Wounds	Categorical to indicate if physical wounds present: yes or no
Latitude	Geographical coordinates (decimal degrees)
Longitude	
Hazard level	Categorical to indicate if hazard is present due to leaning: yes or no
Root Issues	Yes or No
Comments	Additional notes regarding tree health, any unusual characteristics
