# Supplementary material for: Live fast, die young: Accelerated growth, mortality, and turnover in street trees
Source: PLoS One. 2019 May 8;14(5):e0215846. doi: 10.1371/journal.pone.0215846 (PMC6505744; doi:10.1371/journal.pone.0215846)
Supplement: S2 Table — For each of the aggregated study neighborhoods groups, per capita income, standing 2014 biomass, and net carbon balance are noted. Groups are assigned identifying letters in order of standing biomass. (PDF) [file pone.0215846.s002.pdf]

**Neighborhood**	**Group[a]**	**Per Capita Income[b]**	**Street Tree Standing Biomass 2014 (MgC ha^{-1} ± SE)**	**Net C Balance (MgC ha^{-1} yr^{-1} ± SE)**
Hyde Park<br>Roslindale	**A**	$27,978<br>$29,675	8.4 ± 1.2	-0.11 ± 0.15
South Boston<br>South Boston Waterfront	**B**	$40,886<br>$70,913	10.4 ± 1.2	0.08 ± 0.07
Allston<br>Brighton	**C**	$21,018<br>$30,976	12.1 ± 2.1	0.30 ± 0.10
Mattapan	**D**	$21,431	12.3 ± 1.4	-0.50 ± 0.56
East Boston<br>Charlestown	**E**	$22,403<br>$58,566	13.0 ± 1.3	0.09 ± 0.07
Dorchester<br>Roxbury	**F**	$22,120<br>$17,579	13.5 ± 1.9	-0.06 ± 0.13
Back Bay<br>Beacon Hill<br>Downtown<br>North End<br>West End	**G**	$89,658<br>$68,433<br>$59,982<br>$61,765<br>$57,114	14.7 ± 2.5	-2.08 ± 0.43
Fenway<br>South End<br>Longwood Medical Area	**H**	$20,088<br>$57,812<br>$6,376	19.0 ± 2.8	0.01 ± 0.17
Jamaica Plain<br>Mission Hill	**I**	$41,776<br>$20,021	24.3 ± 5.9	-2.02 ± 0.74
West Roxbury	**J**	$39,629	29.2 ± 5.7	-0.72 ± 0.59

[a] Groups determined by proximity and assigned Group letter with increasing biomass. [b] Lima et al 2014. Chinatown, Leather District, and Downtown were combined into "Downtown" for all income-related analyses. The South End and Bay Village were consolidated into the "South End".
