# Supplementary material for: Live fast, die young: Accelerated growth, mortality, and turnover in street trees
Source: PLoS One. 2019 May 8;14(5):e0215846. doi: 10.1371/journal.pone.0215846 (PMC6505744; doi:10.1371/journal.pone.0215846)
Supplement: S3 Table — UEI and 2014 survey data quality methodology. (PDF) [file pone.0215846.s003.pdf]

n	Inconsistency	Resolution	Rationale
293	*Incomplete Entries*	• Eliminated from the final analysis.	Reduce uncertainty.
154	*Oversized Recruits*: Annual growth rates outside the 98th percentile of all 2014 DBHs.	• 2006 DBH was estimated by subtracting the mean genus-level growth rate from the 2014 measured DBH. To avoid bias, these estimations were only included in standing 2006 stock estimates and excluded from the growth analysis.	City of Boston only plants street trees with a DBH of 2 - 2.5 in (5.08 - 6.35 cm)[a]; assumed tree was missed during UEI survey.
54, 29	*Unrealistic Stem and Biomass Growth:* UEI Survey DBH values resulted in unrealistic growth rates.	Trees excluded if<br>• Growth/shrinkage rates were outside of the central 98% of the observed frequency distribution (i.e., > 2.91 or < -2.45 cm yr^{-1}, n=54)<br>• Biomass accrual rates exceeded the 99% central tendency of the distribution (annual C uptake in excess of 930.7 kgC $tree^{-1}$, n=29)	Assumed data entry error. Implemented an overarching data quality assurance method following methods outlined in Rice et al (2004).
2640	*Species/Genus Misidentifications*: UEI Survey assigned incorrect species or genus.	• Used 2014 survey species/genus assignments	All other survey entries were identical for individual (size, location, etc.) and assumed to represent the same tree.

[a] Max Ford-Diamond, personal communication
