# Supplementary material for: Live fast, die young: Accelerated growth, mortality, and turnover in street trees
Source: PLoS One. 2019 May 8;14(5):e0215846. doi: 10.1371/journal.pone.0215846 (PMC6505744; doi:10.1371/journal.pone.0215846)
Supplement: S4 Table — Genus-specific mortality rates ± SE of the 10 most common genera in Boston between 2006 and 2014. (PDF) [file pone.0215846.s004.pdf]

| **Genus** | **Mortality** (% strems yr$^{-1}$) | **DBH growth** (cm stem$^{-1}$ yr) |
|---|---|---|
| *Acer* | 3.66 ± 0.25 | 0.58 ± 0.02 |
| *Fraxinus* | 3.51 ± 0.52 | 1.15 ± 0.04 |
| *Gleditsia* | 1.59 ± 0.32 | 0.99 ± 0.03 |
| *Platanus* | 2.56 ± 0.55 | 0.70 ± 0.06 |
| *Prunus* | 2.69 ± 0.80 | 0.87 ± 0.09 |
| *Pyrus* | 5.60 ± 0.68 | 0.58 ± 0.07 |
| *Quercus* | 3.36 ± 0.82 | 0.85 ± 0.07 |
| *Tilia* | 2.29 ± 0.26 | 0.68 ± 0.02 |
| *Ulmus* | 3.89 ± 0.96 | 1.01 ± 0.07 |
| *Zelkova* | 2.64 ± 0.56 | 1.38 ± 0.05 |
