# Supplementary figures and images for: Live fast, die young: Accelerated growth, mortality, and turnover in street trees
Source: PLoS One. 2019 May 8;14(5):e0215846. doi: 10.1371/journal.pone.0215846 (PMC6505744; doi:10.1371/journal.pone.0215846)

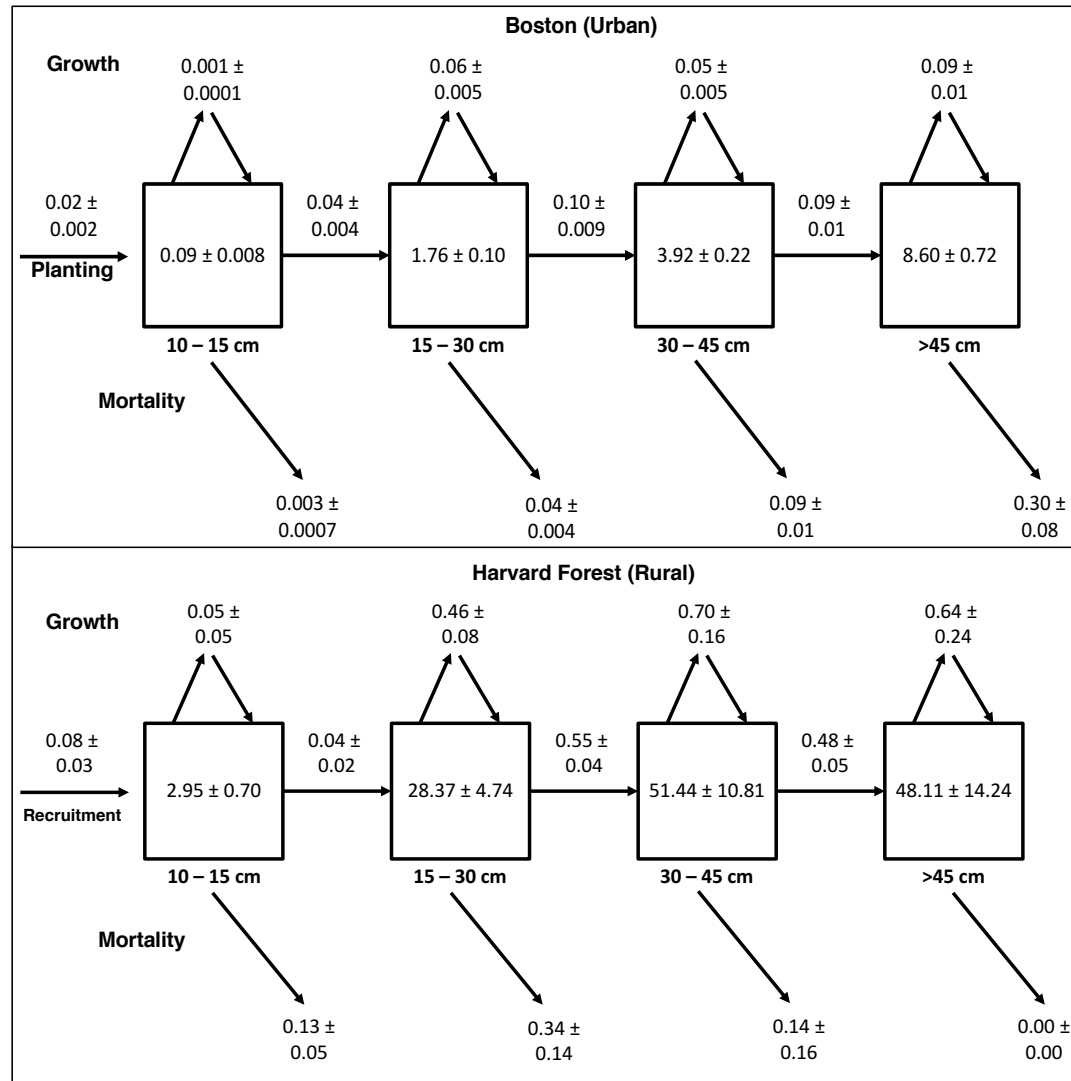

Supplement: S1 Fig — Structure and parameters for box models forecasting short term aboveground carbon pools in Boston street trees (top) and Harvard Forest trees (bottom). Boxes represent pools (Mg C ha-1) and arrows represent fluxes (Mg C ha-1 yr-1). Errors are 95% confidence intervals. (PDF) [file pone.0215846.s005.pdf]
